# Pathological and Surgical Observations on the Diseases of the Joints

**Published:** 1843-08-05

**Authors:** 


*Pathological and Surgical Observations on the Diseases of the Joints.* By Sir Benjamin C. Brodie, Bart., F. R. S., Sergeant-Surgeon to the King, [Queen ?] Surgeon to St. George's Hospital, &c., &c. From the Fourth London Edition, with the Author's Alterations and Additions. Philadelphia: Lea & Blanchard. 1843. 8vo. pp. 216.

It is hardly necessary for us to do more than simply announce the republication of this excellent work of Sir Benjamin Brodie. Its great reputation is perfectly familiar to the surgical student, and it is now placed within his reach in a very neat and readable form. The work has already passed through four editions, and to all the later ones much new matter has been added. Thus several forms of disease are described which are not included in the earlier editions; the descriptions of others have been enlarged; and much that is important and useful with regard to the relief and cure of these affections, which an extended experience dictated, has been suggested. One remark of the author in his preface strikes us as highly important, coming from one whose experience is so ample, and embodying a doctrine but still too much overlooked.

"As I have become more versed in the practical duties of my profession, so I have become more convinced that local diseases, in the strict sense of the term, are of comparatively rare occurrence; and that those which are usually regarded as being of this description may, for the most part, be traced to a morbid condition of the general system. The local treatment of the diseases of the joints, which I now recommend, is even more simple than that which I recommended formerly; but it is quite otherwise with respect to those remedies which operate through the medium of the constitution. Experience has not only confirmed me in the opinion that remedies of this class may often be employed with great advantage to the patient, but has also taught me that there are few cases in which a cure can be obtained without them." *Preface*, p. vi.

The minute morbid appearances of carious joints are, we think, too hastily passed over; no mention is made of the tubercular deposit almost universal in this disease, and so admirably described by M. Nelaton and other French pathologists. Notwithstanding this and some other omissions, the work is the best that exists in any language, and one whose perusal and study will always reward.

